# A New Gastroprotective Effect of Limonoid Compounds Xyloccensins X and Y from *Xylocarpus Molluccensis* in Rats

**DOI:** 10.1007/s13659-014-0034-2

**Published:** 2014-08-03

**Authors:** Vijai Lakshmi, Vaibhav Mishra, Gautam Palit

**Affiliations:** 1 Division of Medicinal and Process Chemistry, Central Drug Research Institute, Lucknow, 226001 UP India; 2 Division of Pharmacology, Central Drug Research Institute, Lucknow, 226001 UP India; 3 Present Address: Department of Biochemistry, King George Medical University, Lucknow, 226003 India

**Keywords:** Gastric ulcer, Xyloccensins X+Y, Antiulcerogenic activity

## Abstract

Gastric ulcer is a very common gastrointestinal disorder affecting a large number of people worldwide. It arises due to an imbalance between aggressive (acid, pepsin and *Helicobacter pylori* infection) and protective (mucin secretion, prostaglandin, epidermal growth factors and bicarbonate) factors in the stomach. In this study, the gastroprotective activity has been investigated of the active constituents from *Xylocarpus molluccensis*. Antiulcer activity of xyloccensins X+Y was investigated and found to be active in various ulcer models in Sprague–Dawley (SD) rats. To understand the mechanism of action of active constituent of natural products, the effects of the compounds on antisecretory and cytoprotective activities were studied. Air dried fruits were extracted with ethanol and fractionated into four fractions. Xyloccensins X+Y were isolated from the active fraction and was tested against different ulcer models. Xyloccensins X+Y were found to possess anti-ulcerogenic activity. The antiulcer activity might be due to its anti-secretory activity and subsequent strengthening of the defensive mechanism. The present study has helped us in identifying a new lead in the form of xyloccensins that could be exploited in the treatment of gastric ulcer disease.

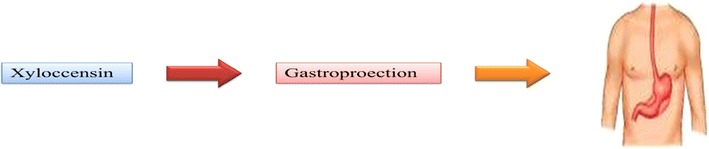

## Introduction

Gastric ulcer is a very common gastrointestinal disorder affecting a large number of people worldwide. It arises due to an imbalance between aggressive (acid, pepsin and *Helicobacter pylori* infection) and protective (mucin secretion, prostaglandin, epidermal growth factors and bicarbonate) factors in the stomach [1]. Stress, smoking, alcohol consumption, *H. pylori* infection and excessive use of non-steroidal anti-inflammatory drugs (NSAIDs) are considered as etiological factors for this disorder [2]. Antacids, proton pump inhibitors, and histamine H_2_ receptor antagonists are commonly used drugs [3, 4]. However, beside their therapeutic efficacies, several incidences of relapse, adverse effects and drug interactions have been shown to be associated with these drugs [5]. Hence, research has been focused on search for new anti-ulcer molecules from medicinal plants as these molecules are more relevant to living system and already have definite biological functions and thus, may have fewer side effects. As a part of anti-ulcer drug discovery programs of our lab several Indian medicinal plants including *Terminalia chebula* [6] *Xylocarpus granatum* [7] and *Nyctanthus arboritristis* [8] have been reported to possess anti-ulcer activity.

*Xylocarpus molluccensis* (Lamk) M.Roem. synonymous *Carapa molluccensis* (Lamk) belongs to Natural Order Meliaceae. It is a mangrove and is commonly known as pussur and Pitakura in Hindi language. It is a tall tree ranging up to 10–12 m tall and trunk of 60 cm diameter at the base, slightly buttressed stem. Bark is red with thick flacks. Wood red in color, leaflets 7–12 × 3–6 cm ovate, acute at apex, oblique at the base, flowers 2–3 cm across, white with red glands inside, stigma cup shaped, fruits 10–15 cm across globose. This species of X*ylocarpus* is uncommon and grows in association with *Heritiera litteralis.* It is mainly found in Mahanadi deltaic region and in Andamans [9]. It has earlier been reported that *X. moluccensis* have been used in the treatment of cholera and fever whereas the fruits are aphrodisiac. The bark as well as pnuematophores of *X. moluccensis* have been reported for neuro pharmacological properties [10] whereas its fruits husk has been reported as bactericidal. The kernels are used in tonics and in relieving colic. The seeds or peels of the fruits are utilized to poultice swellings and ash of the seeds is applied to itch. The fruits are used as a cure for swellings of the breast and in elephantiasis. The bark pressings are used to treat fevers including those caused by malaria. A recent review reveals the various biological activity, chemical constituents and other properties in the *Xylocarpus* genus [11**]**. The most characteristic of the genus *Xylocarpus* are xyloccensins, a class of limonoids. An earlier report from our laboratory revealed the antihyperglycaemic and antidyslipidemic activity in the ethanol extract of *Xylocarpus granatum* fruits in the validated animal models [12]. Keeping in consideration the above facts, we have selected this plant to determine its potential effects on gastric ulcer.

## Results and Discussion

### Effect of xyloccensins X+Y on cold restraint induced ulcer in rats

**Fig. 1:**
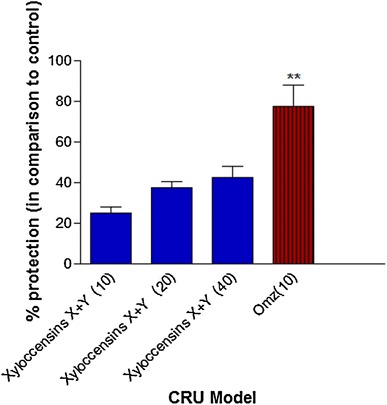
Effect of graded dose of xyloccensins X+Y and reference drug omeprazole (Omz) on percentage protection of ulcer against cold restraint induced gastric ulcer models in rats. Data expressed as mean of  % protection of xyloccensins X+Y and reference drugs after ulcer induction ± S.E.M. Statistical analysis was done by one way ANOVA followed by Dunnett’s Multiple Comparison Test. *Statistically significant at *p* < 0.05 and **p < 0.01, in comparison to control. *n* = 6 in each group

Administration of xyloccensins X+Y at graded doses of 10, 20 and 40 mg/kg, p.o. exhibited 25.0, 37.5, and 42.5 % in CRU model which indicated antiulcer potential of xyloccensins X+Y (Fig. 1).

**Fig. 2:**
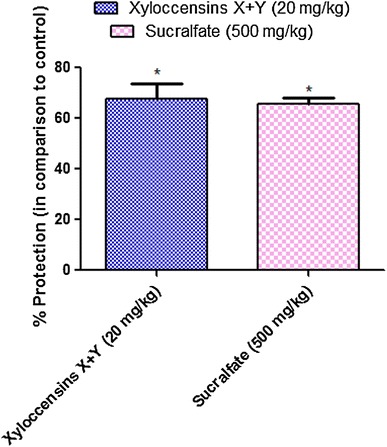
Effect of xyloccensins X+Y and reference drug sucralfate (SUC) on percentage protection of ulcer against alcohol induced gastric ulcer models in rats. Data expressed as mean  % protection of xyloccensins X+Y and reference drugs after ulcer induction ± S.E.M. Statistical analysis was done by one way ANOVA followed by Dunnett’s Multiple Comparison Test. *Statistically significant at *p* < 0.05 and **p < 0.01, in comparison to control. *n* = 6 in each group

### Effect of Xyloccensins X+Y Alcohol Induced Ulcer in Rats

**Fig. 3:**
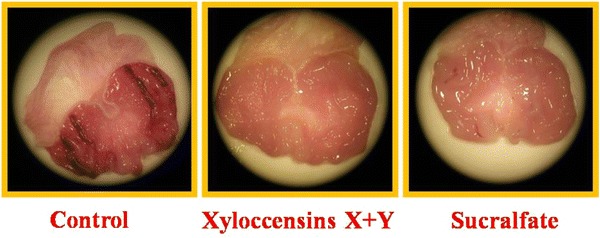
Photographs of control, xyloccensins X+Y treated rats and reference drug sucralfate (SUC) treated against alcohol induced gastric ulcer models in rats

Xyloccensins X+Y showed significant anti-ulcer activity against ethanol induced ulcer showing 67.5 % protection respectively whereas the reference drug, sucralfate (SUC), showed 65.67 % protection as depicted in Figs. 2 and 3.

**Fig. 4:**
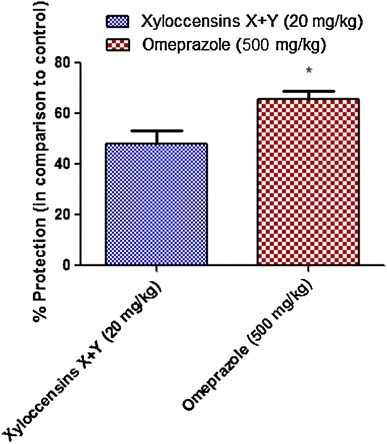
Effect of xyloccensins X+Y and reference drug omeprazole (Omz) on percentage protection of ulcer against pyloric ligation induced gastric ulcer models in rats. Data expressed as mean  % protection of xyloccensins X+Y and reference drugs after ulcer induction ± S.E.M. Statistical analysis was done by one way ANOVA followed by Dunnett’s Multiple Comparison Test. *Statistically significant at *p* < 0.05 and **p < 0.01, in comparison to control. *n* = 6 in each group

**Fig. 5:**
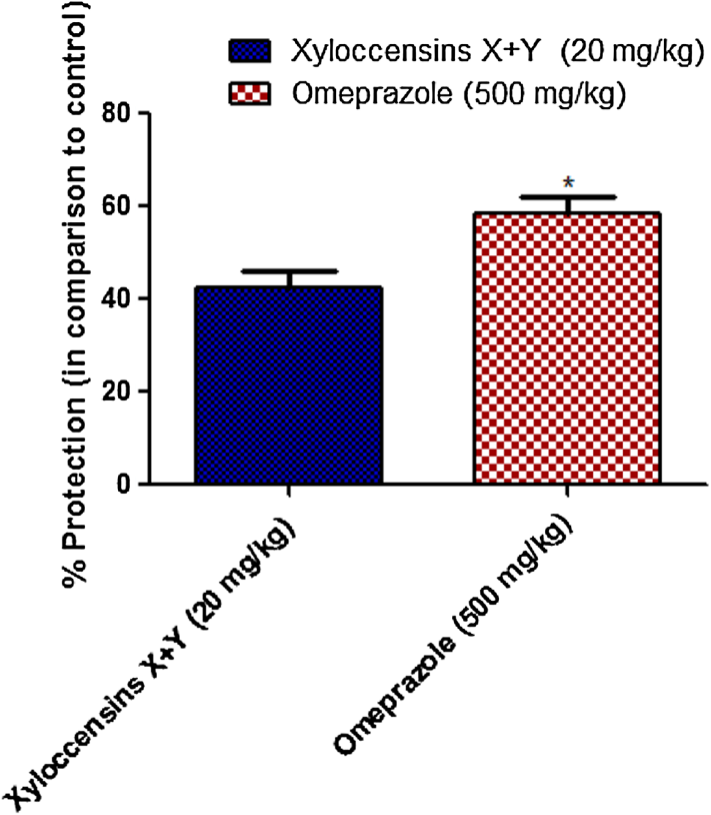
Effect of xyloccensins X+Y and reference drugs omeprazole (Omz) on percentage protection of ulcer against aspirin induced gastric ulcer models in rats. Data expressed as mean  % protection of xyloccensins X+Y and reference drugs after ulcer induction ± S.E.M. Statistical analysis was done by one way ANOVA followed by Dunnett’s Multiple Comparison Test. *Statistically significant at *p* < 0.05 and **p < 0.01, in comparison to control. *n* = 6 in each group

### Effect of Xyloccensins X+Y on Pyloric Ligation Induced Ulcer in Rats

Anti-ulcer activity of xyloccensins X+Y was also observed against pyloric ligation induced ulcer model in rats where it showed protection of 48.0 % whereas reference drug omeprazole (Omz) showed 65.8 % protection (Fig. 4).

### Effect of xyloccensins X+Y on aspirin induced ulcer in rats

Xyloccensins X+Y showed potential anti-ulcer activity (42.5 % protection) in aspirin induced ulcer model, Fig. 5. Whereas the reference drug omeprazole, showed 58.3 % protection in comparison to control (Fig. 5).

### Effect of Xyloccensins X+Y on Gastric Secretion

As shown in Table 1, treatment with xyloccensins X+Y at a dose of 20 mg/kg body weight reduced the free acidity by 8.37 % and total acidity by 10.74 % respectively. Reference drug omeprazole significantly reduced the free acidity by 53.62 % and total acidity by 37.43 %. Xyloccensins X+Y at a dose of 20 mg/kg body weight increased the mucin secretion by 23.99 % in comparison to control (Table 1).Whereas the reference drug omprazole exhibited 32.13 % up regualtion of mucin in comparison to control.

**Table 1:** Effect of and omeprazole (OMZ) on free acidity, total acidity and mucin contents in pyloric ligation model (*n* = 6 in each group)

Treatment	Free acid µequiv/mL	Total acid µequiv/mL	Mucin µg/mL
Control	70.5 ± 6.20	82.0 ± 5.24	746.00 ± 53.20
Xyloensins X+Y (20 mg/kg)	64.6 ± 2.39	73.19 ± 6.34	981.5 ± 112.33
Omeprazole (10 mg/kg)	32.70 ± 2.65**	51.30 ± 2.09**	1099.08 ± 125.64*

Data expressed as mean of µequiv./mL of xyloccensins X+Y and reference drugs after ulcer induction ± S.E.M. Statistical analysis was done by one way ANOVA followed by Dunnett’s Multiple Comparison Test. *Statistically significant at *p* < 0.05 and **p < 0.01, in comparison to control. *n* = 6 in each group

Our present study demonstrated that xyloccensins X+Y isolated from *Xylocarpus molluccensis* possess remarkable anti-ulcer activity in rats. In India; a large number of herbal extracts are used in folk medicine to treat various types of disorders. Xyloccensins X+Y have been studied against various models of experimentally induced gastric ulcer in order to evaluate its mechanism of action involved in prevention of gastric ulcer.

Xyloccensins X+Y exhibited protection in a dose dependent manner in CRU model. A well-accepted model is CRU for the induction of gastric ulcers, in which peripheral sympathetic activation and increased acid secretion play important roles [13]. In addition, xyloccensins X+Y exerted a protective effect against ethanol-induced gastric lesions. Ethanol damages the superficial epithelial layers and inhibits the release of mucosal prostaglandins and depresses the gastric defensive mechanisms [14]. Xyloccensins X+Y appear to augment the gastric mucosal defense indicating the cytoprotective potentials.

Furthermore, gastric acid is an important factor for the genesis of ulceration in pyloric-ligated model [15]. In this model, auto-digestion of mucosa by gastric acid results in the development of ulcers [16]. Xyloccensins X+Y reduced free and total acidity in this model, which suggests its anti-secretory potency.

The cytoprotective ability of xyloccensins X+Y was evident with increase in mucin content in pyloric ligation model and protection against ethanol induced ulcer model in comparison with the reference drugs. To further substantiate the cytoprotective potency of xyloccensins X+Y, its effect against NSAIDs induced ulcer model was explored. Studies suggest that NSAIDs induce ulcers through their effect on cyclooxygenase enzyme leading to reduced prostaglandin production and increase in acid secretion [16]. Our result demonstrated xyloccensins X+Y significantly increased gastric mucin level and exerted significant protection in ethanol induced gastric lesions in rats.

Though different biological activities of *Xylocarpus molluccensis* have been reported earlier, anti-ulcer mechanism of this plant and its active constituents xyloccensins X+Y has not been reported till date. Our study is the first of its kind to establish the anti-ulcer potential of xyloccensins X+Y.

## Materials and Methods

### Plant Material

Fruits of the *Xylocarpus molluccensis* mangrove were collected and identified by Dr. M.N. Srivastava of the Botany Division, Central Drug Research Institute from South Andaman Coast in the month of March. Specimen sample (voucher specimen number 424) has been identified and preserved in the herbarium of the Botany Division of the Institute, Lucknow, India. Fruits were shade dried and crushed.

### Extraction Fractionation and Isolation of Compounds

**Fig. 6:**
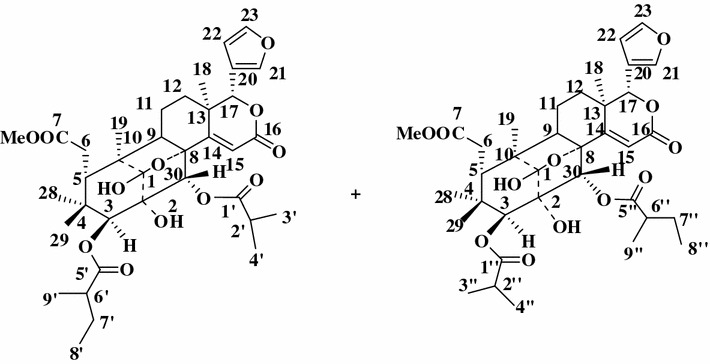
Chemical structures of xyloccensins X+Y

Air dried powdered fruits (1.0 kg) were placed in glass percolator filled with 95 % ethanol 5.0 L and allowed to stand overnight at room temperature, the percolate was collected and the process of extraction was repeated four times. The combined extracts were filtered, concentrated under reduced pressure below 50 °C to minimum volume of 1.0 L. It was further dried in hot air vacuum oven at 50 °C to brown powder (100 g) The brown powder was suspended in water (70 mL) and fractionated into chloroform soluble fraction by extraction with chloroform (5 × 250 mL) in a separating funnel. The combined extract was concentrated under reduced pressure below 50 °C to get brown viscous mass, which was finally dried under high vacuum for 2 h to remove the last traces of solvent (chloroform soluble 25.0 g). The chloroform fraction (20 g) was subjected to silica gel column chromatography (60–120 mesh) and the column (1.2 m length and 4 cm in diameter made of glass was used) was eluted with hexane–ethyl acetate (99:01–0:100) affording 48 fractions (each fractions of 200 mL volume). Fractions showing identical TLC pattern were mixed together and grouped into 6 major groups of fractions. Initial two groups were of very non polar compounds. Rechromatography of fraction 3 (grouped) over silica gel column using hexane–ethyl acetate (99:01–98:02) as eluent and 10 fractions were collected each of 50 mL volume. Fractions showing identical TLC pattern on silica gel plates were grouped together and finally by HPLC reverse phase on C_18_ silica columns using acetonitrile–water 6:4, v/v at 230 nm using uv-detector yielded three compounds namely xyloccensin-E [17] xyloccensin-I [18] and xyloccensins X+Y (Fig. 6). All of these were characterized using IR, nmr, mass, derivatization and comparing the data with those given in literature for these compounds (Xyloccensins E and I). Xyloccensins X+Y were found to be new structures in the isomeric forms [19] (Fig. 6). It was a mixture of two new xyloccensins in the 1:1 ratio on HPLC columns Roy et al. [19] published complete NMR characterization of these compounds in the mixture itself.

### Experimental Animals

All experimental protocols were approved by our Institutional Ethical Committee following the guidelines of CPCSEA (Committee for the Purpose of Control and Supervision of Experiments on Animals) which complies with International norms of INSA (Indian National Science Academy). Sprague–Dawley rats of either sex, weighing 180–200 g were housed in raised bottom mesh cages to prevent coprophagy and were kept in environmentally controlled rooms (temperature 25 ± 2 °C, humidity 60–80 % and 12 h light and dark cycle).

#### Materials and Reagents

Omeprazole and other chemicals were procured from Sigma (St. Louis, MD, USA). Sucralfate was obtained from Meranani Pharmaceuticals, India, whereas all other reagents used were of analytical grade.

#### Treatment Schedule

Graded doses of compounds xyloccensins X+Y (10, 20 and 40 mg/kg p.o.) as well as reference drugs omeprazole (Omz) (10 mg/kg) and sucralfate (SUC) (500 mg/kg) were prepared in 1 % carboxymethyl cellulose (CMC) as suspension and administered orally at a dose of 1 ml/200 g of body weight, 45 min. prior to exposure of ulcerogens. Animals were fasted for 16 h before ulcerogens exposure and were divided into three groups, (*n* = 6).

1. *Group I* (*control*) Control group of animals were treated with vehicle (1 % CMC), 45 min before to exposure of ulcerogens to the animals.
2. *Group II* (*X* *+* *Y treated*) Rats were treated with xyloccensins X+Y (20 mg/kg, p.o.), 45 min prior to the induction of gastric ulcer in all ulceration models.
3. *Group III* (*reference drug treated*) Rats were treated with reference drugs, 45 min. prior to the induction of gastric ulcer in all ulceration models.

#### Anti-ulcer Activity

##### Cold Restraint Induced Gastric Ulcer (CRU)

Animals were subjected to cold restraint stress after 45 min of treatment with compounds or reference drug omeprazole (Omz). Animals in all groups were immobilized in restraint cage and kept at 4 °C in an environmental chamber [20]. After 2 h, animals were sacrificed and stomachs were observed and scored under magnascope for ulcers.

##### Alcohol Induced Gastric Ulcers Model (AL)

Animals for induction of gastric ulcer [21] were given chilled absolute alcohol (1 mL/200 g, body weight). Xyloccensins X+Y and sucralfate (SUC) were administered 45 min before alcohol treatment. After 1 h of alcohol administration, animals were sacrificed and stomachs were cut open along the greater curvature to observe the gastric lesions appearing as hemorrhagic bands along the mucosal ridges of the stomach. Lengths of the lesions were measured using Biovis Image Analyzer software and summated to give a total lesion score.

##### Aspirin Induced Gastric Ulcer Model (AS)

Xyloccensins X+Y and reference drug omeprazole (Omz) were administered 45 min before the treatment of aspirin (150 mg/kg body weight). Animals were sacrificed after 5 h of aspirin treatment and the stomachs were dissected out, incised along the lesser curvature and the lesions were scored [22].

##### Pyloric Ligation Induced Gastric Ulcer Model (PL)

After 45 min of administration of xyloccensins X+Y and omeprazole (Omz), ulcer was induced by pyloric ligation under chloralhydrate anesthesia (300 mg/kg, i.p.). Abdomens were opened and pyloric part of stomach from each rat was ligated avoiding any damage to the adjacent blood vessels [23]. Stomachs were replaced carefully and the animals were allowed to recover with free access to water. After 4 h, animals were sacrificed and stomachs were dissected out. Lesions were scored and gastric fluid was collected and centrifuged at 2000 rpm for 10 min. The supernatant was collected and used for estimation of gastric secretion and mucin level.

#### Gastric Secretion Study in Pyloric Ligation Model

Free and total acidity was measured from the collected gastric juice by titrating against 0.01 N NaOH, using phenolphthalein as an indicator and expressed in terms of μ equiv/mL. Mucin level in gastric juice was quantified as per method reported earlier [24].

#### Ulcer Scoring

Magnascope (5X magnification) were used for ulcer scoring after induction of ulcer via different ulcerogens. Ulcers were scored in all models according to method reported earlier. The severity and intensity of the lesions were graded as following:

(i) Shedding of epithelium = 10
(ii) Petechial and frank hemorrhages = 20
(iii) One or two ulcers = 30
(iv) More than two ulcers = 40
(v) Perforated ulcers = 50.

#### Statistical Analysis

All values shown in the figures and tables represent the mean ± S.E.M. IC_50_ values with 95 % confidence limits were estimated using Maximum Likelihood Iterative Procedure [13]. Statistical analysis was performed with Prism version 3.0 software using one-way analysis of variance (ANOVA) followed by Dunnett’s multiple comparison test. *p* < 0.05 was considered to be statistically significant (*p* < 0.001 = ***, *p* < 0.01 = **, *p* < 0.05 = *, *p* > 0.05 (ns) = not significant).
